# A narrative literature review of the typology of psychiatric emergency services in the UK

**DOI:** 10.1192/bjo.2021.629

**Published:** 2021-06-18

**Authors:** Dhruba Bagchi, George Tadros, Opeyemi Odejimi

**Affiliations:** 1 Birmingham and Solihull Mental Health NHS Foundation Trust; 2 Birmingham and Solihull Mental Health NHS Foundation Trust, Aston University

## Abstract

**Aims:**

This study aims to provide a detailed literature review of the different forms of Psychiatric Emergency Services currently available within the UK.

**Background:**

1 in 6 individuals have one form of mental health disorders. Mental health crisis resulting in an individual requiring access to Psychiatric Emergency Service (PES) can occur at any time. Psychiatric Emergency Service (PES) is described as one that provides an immediate response to an individual in crisis within the first 24 hours. Presently, several PESs are available in the UK with the aim of providing prompt and effective assessment, management and in some cases treatment and/or referral. Over the years, economic and political influences have greatly determined the service delivery models of PES. Indeed, these services vary in name, accessibility, structure, professionals involved, outcomes and many more.

**Method:**

Electronic search of five key databases (MEDLINE, PsychINFO, EMBASE, AMED and PUBMED) was carried out to identify various models of PES in the UK. Various combinations of search terms were used and studies which met the inclusion criteria were selected. Studies were included if they were written in English, conducted within the United Kingdom, and described a form of PES. Search was not limited by years and this is to help have a comprehensive overview as well as show changes over time of the various models of psychiatric emergency services. Studies which did not meet any of the criteria detailed above were excluded.

**Result:**

In total, 59 relevant studies were found which identified nine type of PES-Crisis resolution home treatment, police officer intervention, street triage, mental health liaison services in the Emergency Department, psychiatric assessment unit, integrated services, voluntary services and crisis house. There were more papers describing Crisis resolution home treatment services than the others. Furthermore, majority of the papers reported services within England than other countries within the UK.

**Conclusion:**

All forms of PES are beneficial, particularly to mental health service users, but not without some shortcomings. There is a need to continue carrying out methodological research that evaluate impact, cost-effectiveness as well as identify methods of optimising the beneficial outcomes of all models of PES. This will inform researchers, educationist, policy makers and commissioners, service users and carers, service providers and many more on how to ensure current and future PES meet the needs as well as aid recovery of mental health service users.

